# Competition Triggers Plasmid-Mediated Enhancement of Substrate Utilisation in *Pseudomonas putida*

**DOI:** 10.1371/journal.pone.0006065

**Published:** 2009-06-26

**Authors:** Hiren Joshi, Rachna Dave, Vayalam P. Venugopalan

**Affiliations:** Biofouling and Biofilm Processes Section, Water and Steam Chemistry Division, BARC Facilities, Kalpakkam, India; University of Wisconsin-Milwaukee, United States of America

## Abstract

Competition between species plays a central role in the activity and structure of communities. Stable co-existence of diverse organisms in communities is thought to be fostered by individual tradeoffs and optimization of competitive strategies along resource gradients. Outside the laboratory, microbes exist as multispecies consortia, continuously interacting with one another and the environment. Survival and proliferation of a particular species is governed by its competitive fitness. Therefore, bacteria must be able to continuously sense their immediate environs for presence of competitors and prevailing conditions. Here we present results of our investigations on a novel competition sensing mechanism in the rhizosphere-inhabiting *Pseudomonas putida* KT2440, harbouring *gfpmut3b*-modified Kan^R^ TOL plasmid. We monitored benzyl alcohol (BA) degradation rate, along with GFP expression profiling in mono species and dual species cultures. Interestingly, enhanced plasmid expression (monitored using GFP expression) and consequent BA degradation were observed in dual species consortia, irrespective of whether the competitor was a BA degrader (*Pseudomonas aeruginosa*) or a non-degrader (*E. coli*). Attempts at elucidation of the mechanistic aspects of induction indicated the role of physical interaction, but not of any diffusible compounds emanating from the competitors. This contention is supported by the observation that greater induction took place in presence of increasing number of competitors. Inert microspheres mimicking competitor cell size and concentration did not elicit any significant induction, further suggesting the role of physical cell-cell interaction. Furthermore, it was also established that cell wall compromised competitor had minimal induction capability. We conclude that *P. putida* harbouring pWW0 experience a competitive stress when grown as dual-species consortium, irrespective of the counterpart being BA degrader or not. The immediate effect of this stress is a marked increase in expression of TOL, leading to rapid utilization of the available carbon source and massive increase in its population density. The plausible mechanisms behind the phenomenon are hypothesised and practical implications are indicated and discussed.

## Introduction

Competition between species plays a central role in the activity and structure of communities [1]. Stable co-existence of diverse organisms in communities is thought to be fostered by individual tradeoffs and optimization of competitive strategies along resource gradients [1]. Outside laboratories microorganisms usually coexist in multicellular communities and compete with one another for limited natural resources. This kind of competition between microbes plays a major role in framing the community structure and, in turn, helps proliferation of certain species in a given niche, where appropriate strategies help it to outcompete others [2]. The strategies include production of various antibiotics, toxins, exo-enzymes, siderophore-like molecules and prophage induction [3], [4]. Amongst others, plasmid acquisition is also known to be employed by microbes while adapting to a new environment. Acquiring plasmid is a gain of function but there is biological cost associated with it, in terms of energy burden, which might make the organism less competitive [5]. Thus, the modulation in the expression of the plasmid-coded genes under specific environmental conditions becomes a necessary strategy for better survival [5]. It is widely demonstrated that plasmid replication and expression are dependent on various environmental stresses, including amino acid starvation, heat and cold shock responses, salt concentration, pH, etc. [6]. Although extensive research has been carried out to study the biochemistry of replication, copy number maintenance and partitioning of plasmids, there are few reports focusing on the mechanisms underlying modulation of plasmid expression in response to environmental stimuli [6]. Similarly, the role of plasmids in competitive survival strategies is less researched, except for a few reports where models have been developed for competitive behaviour of plasmid-bearing and plasmid-free organisms in fermentors and bioreactors [7]–[9].

In natural environment, microorganisms are bound to come in contact with one another. Initially, the microbes may live by mutual co-operation, but, at a stage when local concentrations are high or resources are limited, competitive interactions do occur [2]. For example, quorum sensing molecules that coordinate and reinforce community behaviour in many bacterial species are released and are recognized by bacteria and used for competitive survival [2]. Another competitive strategy possibly employed by bacteria is recognition by physical contact. Physical contact allows bacteria to recognize a competitor, the immediate benefit being ability to rapidly mobilise mechanisms to utilise available resources, thereby outcompeting the competitor. Although the former phenomenon is very well characterized, the latter method of sensing competition is hardly discussed and needs to be investigated in detail.

Being a root colonizer and habitant of extremely competitive rhizosphere environment, *Pseudomonas putida* is extensively studied in terms of its role as bio-control agent, in bioremediation and its survival in rhizospheric environment. Interestingly, it is a natural host for many plasmids which are involved in organic compound degradation [10], which help it to survive in polluted environments. It was thought that *P. putida* harbouring TOL plasmid (pWWO) would be a good model system to study plasmid-mediated competitive interactions in mixed consortia. Plasmid pWWO consists of genes responsible for meta-degradative pathway of toluene and benzyl alcohol (BA). It also consists of GFP under regulation of the same promoter. Thus, any enhanced expression of pWWO can be marked with rapid BA utilization and superior GFP fluorescence [11]. In analysing the problem, the specific questions we raised were: (a) how would *P. putida* respond to the presence of another species, which might be a potential competitor for the resources? (b) How does it sense the presence of competitors? (c) Once in the competition race, does it modulate its plasmid expression for its own advantage? In this paper we examine these issues and demonstrate using microcosm experiments that planktonically growing *P. putida* senses presence of competitors by physical cell-cell interactions and not by means of diffusible substances and that such sensing is followed by enhanced expression of genes on plasmid pWWO, aiding rapid utilisation of the available carbon sources and massive increase in its population density.

## Results

**Figure 1:**
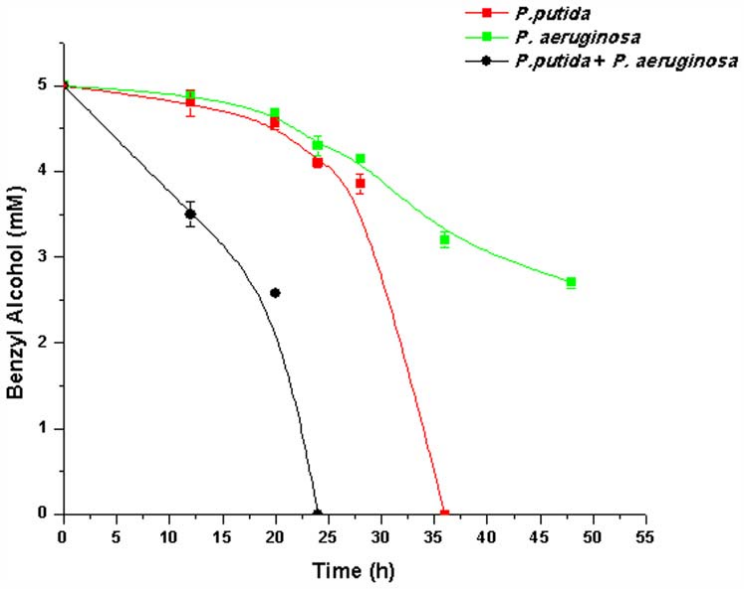
Time course of BA degradation by PP9, PAO1 and PP9+PA01. PP9 and PAO1 (10^6^ cells each) and PP9+PA01 (10^6^+10^6^) were inoculated in Tris+BA medium and incubated at 100 rpm and 30°C. Samples were withdrawn at regular time intervals and the residual BA was estimated by HPLC.

Comparative analysis of time course of benzyl alcohol (BA) utilization by *Pseudomonas putida* KT2440 (termed PP9) and *Pseudomonas aeruginosa* (PAO1) was done using monospecies and two-species cultures. Both the species are BA degraders. About 10^6^ cells each of PP9 and PAO1 were inoculated separately in shake flasks containing 100 ml Tris minimal media containing 5 mM BA as the sole carbon source. Samples were drawn at different intervals and analyzed for residual BA. As shown in Figure 1, PP9 utilized 50% of BA in 30 h; the organism completely utilized BA in 36 h, whereas PAO1 utilized 50% BA in 48 h. In order to find out how the two organisms, both having the ability to utilize the same carbon source, do when cultured together, we inoculated 10^6^cells each of PAO1 and PP9 together in 100 ml Tris BA medium. The time-course of BA utilization by the dual-species consortium is shown in Figure 1. BA was utilised to the extent of 50% in 17 h and completely in less than 24 h of incubation. A significant reduction in the shoulder indicated that the degradation started earlier than in the previous case (PP9 alone), with reduced induction time.

**Figure 2:**
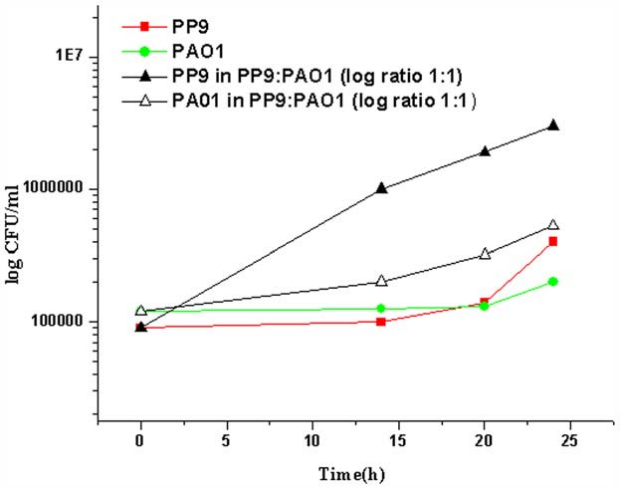
Plate count of PP9, PAO1 and PP9+PAO1 grown as mono and dual cultures. PP9 (10^6^ cells), PAO1 (10^6^ cells) and PP9+PA01 (10^6^ cells each) were inoculated in Tris+BA medium and incubated at 100 rpm and 30°C. Samples were withdrawn at regular time intervals and the samples were plated in respective antibiotic containing media for enumeration.

It is possible that enhanced degradation was a result of combined action by the two degraders. However, if one of the two were to degrade the sole carbon source more competitively than the other, one would expect its number to go up in the culture. To investigate whether or not a particular organism flourished better when co-cultivated, we performed bacterial counts of the individual organisms at various time intervals. We plated samples of the mixed species consortium on luria agar containing gentamicin 25 µg.ml^−1^ (to count PAO1) and luria agar containing kanamycin 30 µg.ml^−1^ (to count PP9). As can be seen from the results, PP9 outnumbered PAO1, indicating that the former numerically outcompeted the latter (though both are BA-degraders) when the two were grown together (Figure 2).

**Figure 3:**
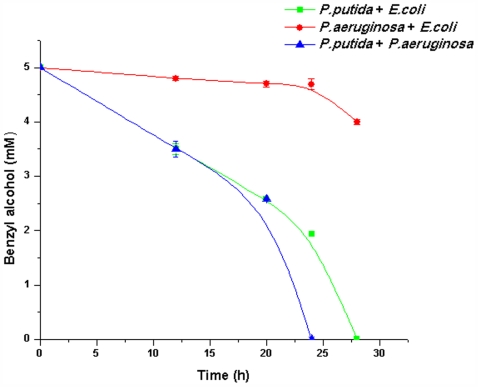
Time course of BA degradation in co-cultures of PP9+*E. coli* JM 101, PAO1+*E. coli* JM 101 and PP9+PAO1. 10^6^ cells each of PP9+*E. coli* JM 10, PAO1+*E.coli* JM 101and PP9+PAO1were inoculated in Tris+BA medium and incubated at 100 rpm and 30°C. Samples were withdrawn at regular time intervals and the residual BA was estimated by HPLC.

The above results indicate that PP9 was able to utilise BA much more efficiently when it was grown as a co-culture rather than as a monoculture. To further confirm whether the faster resource BA utilization by PP9 is triggered by the presence of another organism and whether PP9 resorts to enhanced BA degradation only when the counterpart has the ability to utilize the same carbon source, we utilized the BA non-degrader *E. coli* JM101 in place of PAO1 in the dual species experiments. Cells of *E. coli* JM 101 and PAO1 (10^6^ each) and PP9 and *E. coli* JM 101 (10^6^ each) were inoculated in shaking flask containing Tris minimal media with 5 mM BA. The BA utilization rate of PP9: *E. coli* JM101 (degrader and non-degrader) was similar to the BA utilization rate of PP9: PAO1 (both degraders) (Figure 3). The former dual culture utilized BA completely in 27 h whereas the latter did it in 24 h. Interestingly, the results indicated that, when *E. coli JM101* (non-degrader) was inoculated along with PAO1 (degrader sans the plasmid), there was no increase in the BA utilization compared to when *E. coli* JM 101 was used together with PP9 (Figure 3), indicating that enhanced degradation takes place only in the case of the plasmid-bearer. Moreover, after 30 h the PAO1: *E. coli* JM101system had only utilized just 4% of the initial BA.

From the above results one may infer that the presence of another organism (be it BA degrader or non-degrader), is perceived by PP9 as competition, thereby leading to enhanced expression of TOL and rapid utilization of BA. To investigate the mechanistic aspect of competition sensing by PP9, two hypotheses were proposed: PP9 senses competition 1) with the help of diffusible signalling molecules released by the competitor or 2) by physical collision between the two species.

To test the first hypothesis, the 15 h old co-culture of PP9+*E. coli* JM 101 (log ratio 1∶1) growing at 30°C in Tris BA media was centrifuged at 5000 rpm. The supernatant obtained was filtered through 0.22 µm filter to remove any bacteria left behind and the filtrate (conditioning media) was subsequently inoculated with 10^6^ cells of PP9 and PP9: *E. coli* JM 101 (log ratio 1∶1). Before inoculation, the BA in the filtrate was estimated by HPLC and fresh BA was added to make up to a final concentration of 5 mM. In a similar way, 10^6^ cells of PP9 and PP9: *E. coli* JM 101 (log ratio 1∶1) were inoculated in fresh Tris BA medium. The flasks were incubated on a shaker at 100 rpm and 30°C and BA degradation profile was monitored and compared (data not shown). Results showed that the BA utilization rate in the conditioned medium inoculated with PP9 was similar to that by PP9 alone as a monoculture in fresh medium. On the other hand, BA degradation profile by PP9: *E. coli* JM 101(log ratio 1∶1) in conditioning media was also similar to that observed in fresh Tris BA media, also suggesting that the conditioning media did not contain any metabolites or any micronutrient deficiency that hindered the growth.

**Figure 4:**
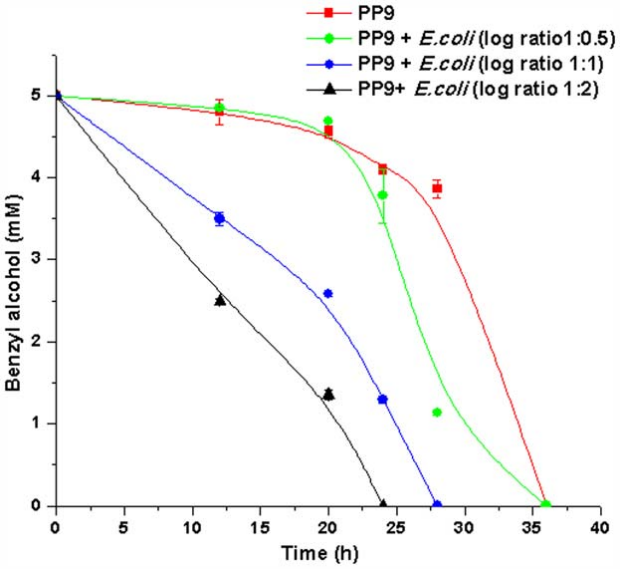
Time course of BA degradation by PP9 in presence of increasing *E. coli* JM101 cell density. PP9 and *E. coli* JM 101 were inoculated in Tris BA medium along in different log ratios (1∶0.5, 1∶1 and 1∶2) and incubated at 100 rpm and 30°C. Samples were withdrawn at regular time intervals and the residual BA was estimated by HPLC.

**Figure 5:**
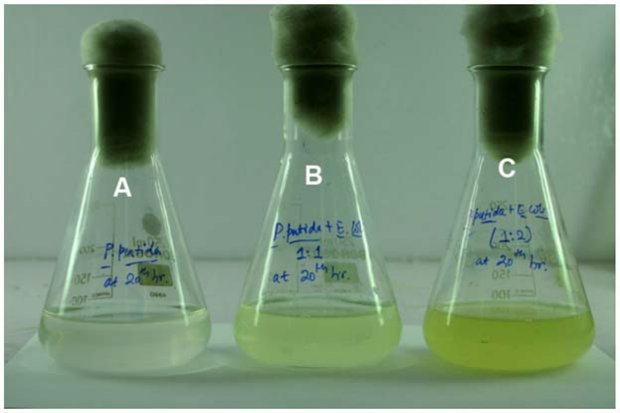
Shake flasks showing differences in GFP expression (after 20 h incubation) in PP9 cells grown in presence of difference cell densities of *E. coli* JM101 Blue counterpart. (A) 10^6^ cells of PP9 were inoculated in 100 ml Tris BA medium, (B) 10^6^ cells of PP9 and 10^6^ cells of *E. coli* JM101 inoculated in 100 ml Tris BA, (C) 10^6^ cells of PP9 and 2×10^6^ cells of *E. coli* JM101 inoculated in 100 ml Tris BA. All flasks were incubated at 100 rpm and 30°C. After 20 h, 9% BA was utilized by PP9 in Flask A, whereas in flask B and C, PP9 utilized 52% and 73% BA respectively.

**Figure 6:**
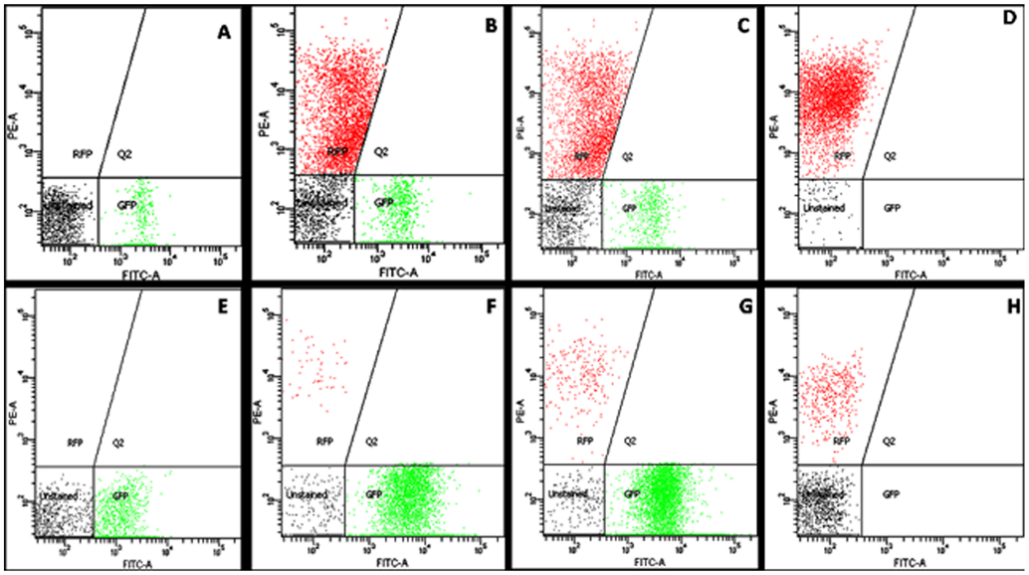
FACS analysis of GFP expression in PP9 cells grown in presence and absence of *E. coli* XL-1 Blue counterpart. FACS and cell counts analyses were carried out as described in methods. The relative fluorescence is indicated on the x and y axes with the filters as shown. The X-axis shows PP9 cells expressing GFP and the Y-axis shows the *E. coli* XL-1 Blue cells expressing DsRed. (A–D) PP9, PP9: *E. coli* XL-1 Blue (log ratio 1∶1), PP9: *E. coli* XL-1 Blue (log ratio1∶2) and *E. coli* XL-1 Blue at 0 h; (E–H) PP9, PP9: *E. coli* XL-1 Blue (log ratio 1∶1), PP9: *E. coli* XL-1 Blue (log ratio 1∶2) and *E. coli* XL-1 Blue after 24 h.

**Figure 7:**
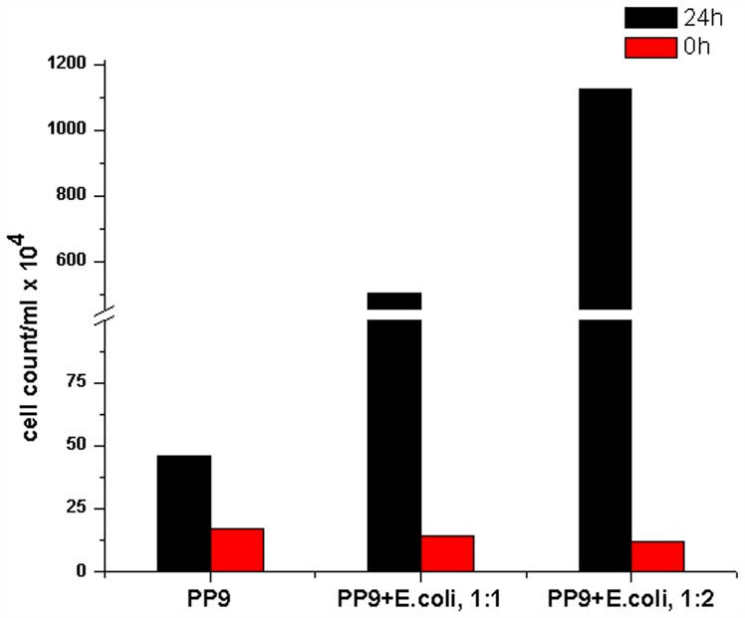
Cell counts of GFP expressing PP9 cells in presence and absence of *E. coli* XL-1 Blue counterpart obtained from FACS.

Based on the above results, it was further hypothesised that physical collision between cells might be playing a role by being recognized as presence of competing cells by PP9. As the cell number of the competing cells increased, physical collision would also increase, prompting PP9 to hasten BA utilization. To validate this, PP9 was inoculated with increasing cell density of *E. coli* JM 101 (PP9: *E. coli* JM101 in the log ratio 1∶0.5, 1∶1 and 1∶2) in Tris+BA medium. BA utilization was found to be the highest in the case of PP9: *E. coli* log ratio of 1∶2, followed by 1∶1 and the least in 1∶0.5 (Figure 4). The data clearly indicated that increasing cell density of the “competing” counterpart resulted in increasing BA utilisation by PP9. This could have been achieved by means of an increase in the expression of proteins responsible for BA utilisation. We observed an increase in GFP expression (putatively, a consequence of enhanced TOL expression) in response to increased “competitor” density in the shake flasks (Figure 5). To further establish that the presence of another strain in the mixed culture leads to enhanced expression of TOL in PP9, we performed flow cytometry experiments. For FACS analysis, instead of *E. coli* JM 101 (used in the previous experiments) we have used DsRed-bearing *E. coli* Strain XL-1 Blue (see Table 1) to differentiate and count the populations of PP9 and *E. coli*. Cultures of PP9, PP9: *E. coli* XL-1 Blue (log ratio 1∶1) and PP9: *E. coli* XL-1 Blue (log ratio 1∶2) were grown in presence of 5 mM BA and analysed after 0 h and 24 h (Figure 6 and 7). At the end of 24 h, the GFP positive cells in PP9 alone culture had increased by 3 fold, whereas in the PP9: *E.coli* XL-1 Blue (log ratio 1∶1) and PP9: *E. coli* XL-1 Blue (log ratio 1∶2) cultures, the increase was 34 and 87 fold, respectively (Figure 7). The FACS results lend credible support to our contention that enhanced BA degradation is mediated via TOL plasmid activation, which in turn, is related to the cell density of the “competing” species. Seen together with data presented in Figure 2, the FACS results unequivocally show the tremendous competitive edge derived by PP9 by employing this kind of strategy.

**Figure 8:**
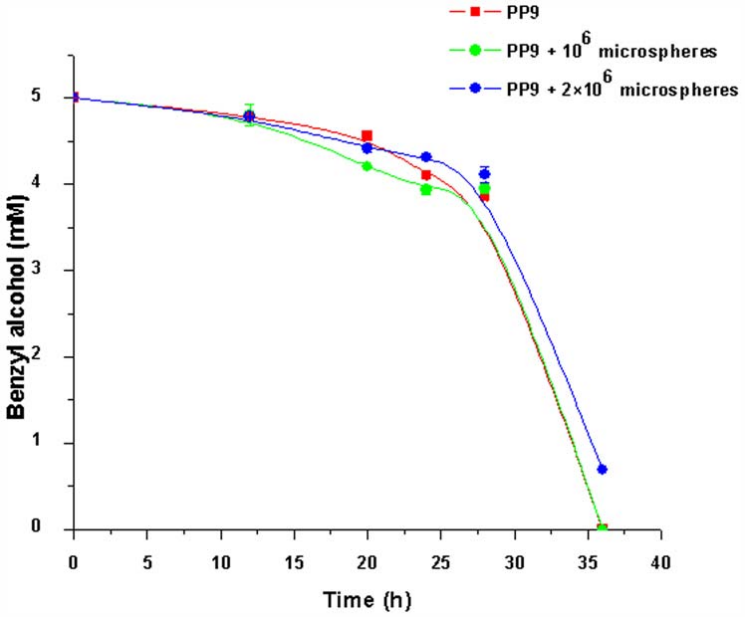
BA degradation profile by PP9 in presence of inert microsphere mimicking competitor. 10^6^ cells each of PP9 were inoculated with 10^6^ and 2×10^6^ microspheres (size: 0.5 µm) in Tris BA medium and incubated at 100 rpm and 30°C. Samples were withdrawn at regular time intervals and the residual BA was estimated by HPLC.

The next question we asked ourselves was whether the triggering was merely the outcome of physical contact and, if so, whether such mechanosensitive stimulation could be achieved by using inert particles that mimicked competitor cells. To mimic the role of the *E. coli* JM 101 cells in triggering the enhanced degradation of BA and to investigate whether the role of the counterpart is merely physical, we used 0.5 µm polystyrene microspheres (Polysciences USA) [please note that the *E.Coli* JM 101 cells are ∼0.5 µm in diameter [12]]. The experiments were carried out as earlier. PP9 (10^6^ cells) was inoculated with the microspheres (10^6^ and 2×10^6^ particles) in 100 ml Tris BA medium. As shown in Figure 8, there was no significant increase in degradation of BA by PP9 in the presence of microspheres. Moreover, increasing the number of microspheres did not have any effect on the degradation rate. This observation clearly established that simple physical interaction could not have caused the increase in BA utilisation by PP9.

**Table 1:** List of bacterial strains used in the present study.

Strain/Plasmid	Description	Reference
*Pseudomonas putida* KT2442	TUM-PP9 (pWWO::gfpmut3b); Rifr Km^r^ Nal^r^	[11]
*Pseudomonas aeruginosa*	PAO1 (las R); Gen^r^	[23]
*Escherichia coli* JM101	thi-1 Δ(lac-proAB)	Present study
*Escherichia coli* XL-1 Blue	recA1 endA1 gyrA96 thi-1 hsdR17 supE44 relA1 lac [F' proAB lacIqZΔM15 Tn10 (Tetr)]	[22]
*Lactobacillus* sp.	Natural isolate	Present study
*Deinococcus radiodurans*	Wild type	[24]
**Plasmids**		
TOL-gfpmut3b (pWW0)	TOL-gfpmut3b Integration of PA1/04/03Dgfpmut3b from pJBA28 into TOL	[11]
pDG75	DsRed	[22]

**Figure 9:**
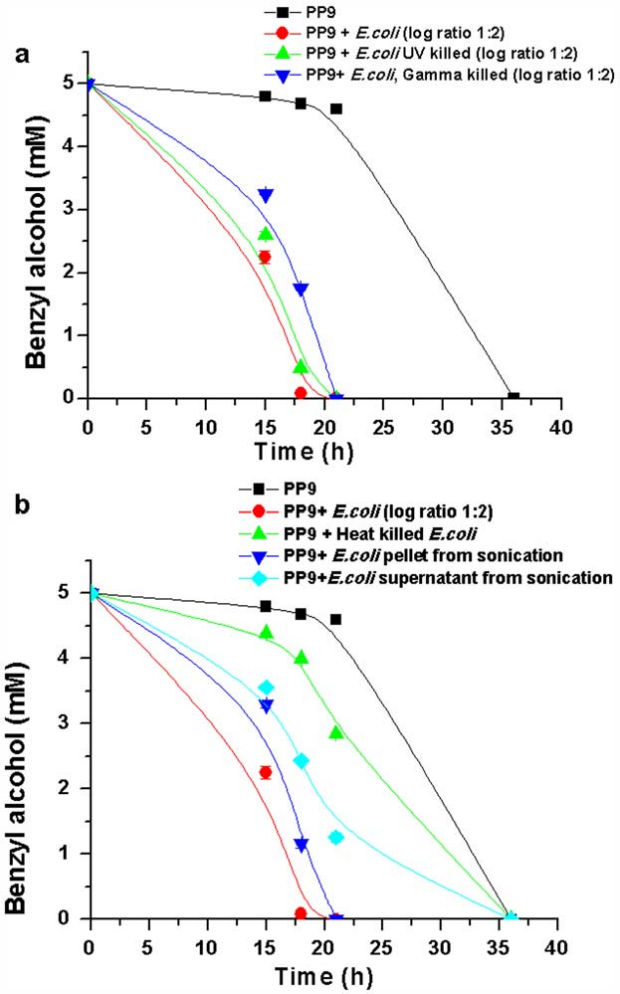
Results of experiments using killed competitor cells. (9a) 10^6^ cells of *E.coli* JM101 were killed by UV and gamma rays and inoculated with live PP9 (10^6^ cells) in Tris BA medium and incubated at 30°C and 100 rpm. Samples were withdrawn at regular intervals and residual BA was estimated with HPLC. (9b) Results of experiments using killed competitor cells. 10^6^ cells *E. coli* JM101 was subjected to heat killing and sonication and inoculated with live PP9 cells (10^6^) in Tris BA medium at 30°C and 100 rpm. Samples were withdrawn at regular intervals and residual BA was estimated with HPLC.

In order to verify whether receptor-ligand kind of interaction could be involved in the induction phenomenon, experiments were repeated with dead and cell wall compromised competitor cells. Different cell killing methods having variable impact on cell wall integrity were used to kill the competitors. UV and gamma exposure were used as cell killing methods that mostly targeted the DNA, while boiling at 100°C for 15 min and sonication (at 20 KHz) were employed as techniques that would impair cell wall integrity. *E. coli* (2×10^6^ cells) were exposed to different cell killing methods and the killed cells (after viability check) were inoculated along with 10^6^ cells of live PP9 in Tris BA medium and BA degradation was monitored. Additionally, *E. coli* JM 101 cells were also sonicated at 20 KHz. The culture was centrifuged and divided into two fractions: soluble fraction (supernatant) and cellular fraction (pellet), which were further inoculated in two separate flasks containing 10^6^ cells of PP9. The results (Fig. 9a) indicate that the three cultures (PP9+intact *E. coli* JM101; PP9+UV killed *E. coli* JM101 and PP9+Gamma killed *E. coli* JM101.) showed similar BA degradation profile, which as much faster than that of *P. putida* alone. Figure 9b, however, shows that BA degradation rate was in the decreasing order of PP9+*E. coli* JM101>PP9+*E. coli* JM101 cell wall fraction>PP9+*E. coli* JM101cell lysate fraction>PP9+*E. coli* JM101 heat-killed cells. The marginal induction by the cell lysate could be attributed to the remnants of the cell wall fraction present in the supernatant. These results further affirm the role of unknown cell wall component(s) in the sensing of competition by PP9 thereby, leading to higher TOL expression and faster BA utilization.

**Figure 10:**
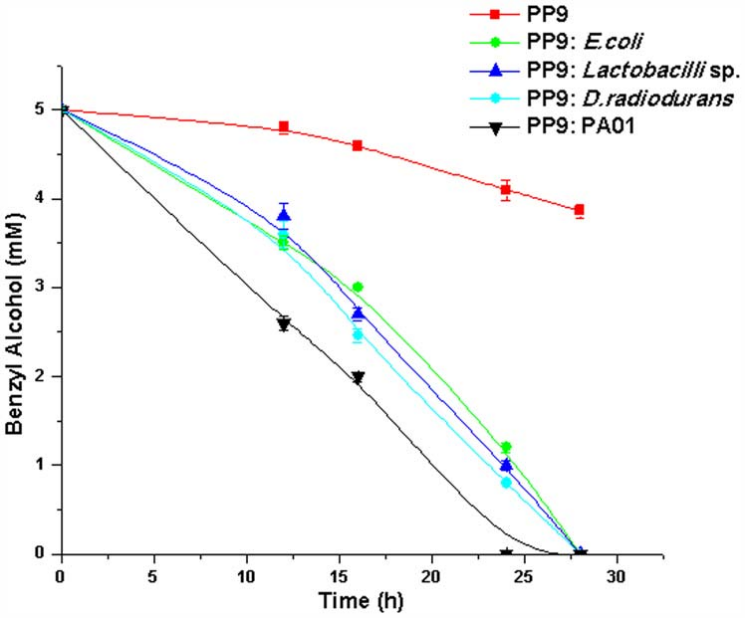
Time course of BA degradation by PP9 in presence of different bacteria. 10^6^ cells of PP9 were inoculated with similar number of *Lactobacillus* sp., *Deinococcus radiodurans*, *P. aeruginosa* PAO1 and *E. coli* JM 101. Samples were withdrawn at regular intervals and residual BA was estimated with HPLC.

Lastly, to check whether enhanced BA utilization in PP9 is only induced in presence of PA01 and *E. coli* JM 101 or whether this phenomenon is more generalized and stands true for other bacterial species as well, we monitored BA degradation profile of PP9 in mixed cultures containing a Gram positive bacillus (*Lactobacillus* sp.) and a Gram negative coccus (*Deinococcus radiodurans*) (both are non BA degraders). The experiments were repeated with PP9 and the other two bacteria in the log ratio of 1∶1 each. As shown in Figure 10, BA degradation was found to be enhanced in all the cases where PP9 was co-cultured with another bacterial species. The results, thus, indicate that competition stress emanating from multiple sources can induce PP9 to enhance its TOL expression.

## Discussion

Competition for substrate is considered one of the major driving forces in bacterial interactions and numerous published data have shown how bacteria in different conditions effectively outcompete others because of better utilization of limited energy resources. The primary requirement for competitive fitness lies in early detection of competition and mounting an appropriate response to it in the shortest time possible. Bacteria have evolved various sophisticated cell-cell communication systems which enable them to assess presence of potential competitors or mating partners. This mainly involves secretion and detection of auto-inducers and quorum sensing molecules [13]–[15]. The major bottleneck of this sensing mechanism is that the competition can only be sensed when the competitor releases molecules and the counterpart possesses appropriate receptors to sense the ligand. Aoki *et al* [16] have demonstrated novel mechanism of contact-dependent growth inhibition (CDI) in *E. coli*, where one strain of *E. coli* inhibits another by signaling through physical contact. In this case, the competitive strategy involves inhibiting the growth of other microorganisms. Similarly, contact-dependent signaling is known in *Myxococcus xanthus* fruiting body development [17]. Such work has initiated new directions where microbes have been shown to interact by physical contact, apart from well established quorum sensing and auto-inducer mediated signalling, mediated via diffusible molecules. However, most of the contact-dependent signalling has been shown to be between the same species. Here we demonstrate that contact-mediated response to competition could take place between different species. Significantly, the organism attains competitive edge not by inhibiting the competitor, but by increasing its own metabolic function, such that it rapidly utilises the available carbon source and multiply in number. The results look all the more interesting as they indicate enhanced TOL expression by PP9 even when the “competing” species (in the present case, *E.Coli* JM101) is incapable of metabolising the given carbon source, which means that PP9 cannot differentiate between a substrate degrader and a non-degrader. However, PP9 could differentiate between a live (potential) competitor and inert particles, and when encountered with the latter, does not alter TOL expression. Our experimental results with *Lactobacillus* sp. and *D. radiodurans* show that the mechanism is also active against different types of bacteria. At this stage it is not clear if this kind of competition-induced response is also exhibited by other plasmid-bearing strains. Nevertheless, the findings may have practical applications, wherein one may be able to speed up substrate conversion (e.g., in a bioreactor) by a given strain by simply adding another strain (actually, a non-competitor) incapable of using the same substrate. The second strain may be chosen in such a way that it does no more than makes its presence felt and does in no way interfere with the activity of the first one.

To summarise, our results suggest that *P. putida* can sense competition by contact-dependent processes involving unknown ligand-receptor type of interaction. The response is elicited only when live or cell wall-intact cells are used and inert particles do not elicit any competitive response. Based on the results of the experiments involving different strains (both gram positive and gram negative) of bacteria, we infer that the observed phenomenon of physical contact-dependent induction of plasmid expression is generalized (i.e., active against many types of bacteria) and is non-specific in nature. Such a strategy provides a definitive competitive edge to the organism displaying it. We further hypothesise that the above strategy may be more efficient than the one based on detection of possible diffusible molecules secreted by the competitor, since the lead time advantage would be considerable. We need to carry out similar studies using other plasmid-bearing species in order to conclude whether the phenomenon is widely prevalent among bacteria.

At this stage we are not in a position to offer a mechanistic explanation for the contact-dependent enhancement of expression exhibited by PP9. Our current hypothesis is that the cell-cell interaction could be mediated through cell wall structures such as pili or flagella. The PP9 strain employed in the present work is a motile organism possessing monotrichous flagellum [18]. It has been shown that bacteria use flagella to sense their external milieu [19]. Our future experiments will look at sensing of competition by de-flagellated (mechanically or mutationally) cells and by those cells residing in biofilms. The latter are known to shed flagella upon switching from planktonic to biofilm mode of life [20]. Alternatively, it is possible that lectin type of interactions [21] could be involved in tactile recognition of a competing strain. Such interactions could be probed with the help of specific blocking agents [21].

## Materials and Methods

### Strains and growth conditions

The experiments were carried out using *Pseudomonas putida* KT2440 (represented as PP9), harbouring *gfp*mut3b-modified Kan^R^ plasmid pWWO. Plasmid pWWO was used since it is a well-characterized plasmid that codes for the degradation of toluene and benzyl alcohol. Other bacterial strains used in various experiments and their relevant characteristics are listed in Table 1. Benzyl alcohol (BA) (Merck, Germany) was used as the sole carbon source in competition experiments. Among the strains used, PP9 and PAO1 are capable of BA utilisation, while *E.coli* JM101, *Lactobacillus* sp. and *Deinococcus radiodurans* is a non BA degrader (experimentally determined). All bacterial strains were grown in a minimal medium that consisted of the following (grams per litre of H_2_0): Tris, 6.05; Sodium chloride, 4.67; Potassium chloride, 1.5; Ammonium chloride, 1.06; Sodium sulphates, 0.42; Magnesium chloride, 0.233, Calcium chloride, 0.03; Sodium dihydrogen phosphate (dehydrate), 0.004. A stock solution of trace elements was prepared that contained the following (in mg/l): Zinc sulphate (heptahydrate), 143.77; Magnesium chloride (Tetrahydrate), 98.96; Boric acid, 61.83; Cobalt chloride, 190.34; Copper chloride, 17.05; Nickel chloride, 23.77; and sodium molybdate, 26.29. From the trace elements solution 100 µl was added to the above Tris solution. Benzyl alcohol, 5 mM was added as the sole source of carbon.

### Shake flask experiments

All experiments were conducted in 250 ml flasks containing an initial concentration of 5 mM BA in 100 ml Tris minimal medium (pH 7.5) at 30°C, 100 rpm. About 10^6^ cells were inoculated. Samples were withdrawn at regular time intervals for HPLC analysis and microscopic observations.

### CFU measurements

For CFU measurements, 10^6^ cells each of PP9 (Kan^r^), PAO1 (Gen^r^) and PP9 (Kan^r^)+PAO1 (Gen^r^) (10^6^+10^6^) were inoculated in Tris+BA medium and at regular intervals samples were plated on luria agar+kanamicin (30 µg.ml^−1^) and luria agar+gentamicin (25 µg.ml^−1^) plates for CFU measurements.

### FACS analysis

Flow cytometry experiment was carried out to show that presence of a “competitor” induces PP9 to express proteins coded on the TOL plasmid and increase its number by faster substrate utilisation. Fluorescence assisted cell sorter was used to differentiate between populations of PP9 harbouring GFP and *E. coli* XL-1 Blue strain harbouring DsRed [22]. The experiments were carried out using a BD Biosciences FACS Aria II instrument with 70 µm sorting nozzle at low pressure. GFP-mut3 and DsRed were excited using a 488 nm blue laser and detected using 530/30 nm and 616/23 nm filters, respectively. Culture samples (0 and 24 h) of PP9, *E.coli* XL-1 Blue, PP9: *E.coli* XL-1 Blue (log ratio 1∶1) and PP9: *E.coli* XL-1 Blue (log ratio 1∶2) was used in the experiments. PP9 cells expressing GFP in response to increasing competitor cell density were counted using the BD FACSDiva software provided by the manufacturer.

### HPLC analysis

Samples for HPLC were centrifuged at 8000 rpm for 10 min and the amount of BA in the supernatant was estimated with a Waters (Milford, Massachusetts, USA) HPLC using a Bondapak C18 reverse-phase column (Waters, Milford, Massachusetts, USA) and a UV-Visible detector set at 256 nm. The mobile phase was a mixture of 95% water (Milli Q, Sartorius Ultra Pure, Goettingen, Germany) and 5% acetonitrile (HPLC Grade; Merck) pumped at a flow rate 1.3 ml.min^−1^. The HPLC chromatogram displaying the overlap of three injections, 5 mM BA in Tris Medium is shown in Supporting Information (Figure S1).

### Cell killing experiments

Experiments involving dead cells were performed using different cell killing treatments, namely, exposing the bacteria (2×10^6^ *E. coli* cells) to UV light (irradiated at the rate of 3.5 J/m^2^/sec with 254 nm UV light, Philips TUV 30W) for 30 min, irradiation to lethal radioactivity (500 Gy, dose rate of 4.5 KGy/h using ^60^Co source) and boiling for 15 min in a boiling water bath. In all cases, loss of viability in treated cells was confirmed by plating. To obtain cell wall fraction and cell lysate, 2×10^6^ *E. coli* cells were sonicated (Sonics, Vibrocell, CT, USA) on ice at 20 KHz power level for 20 min, followed by centrifugation at 8000 rpm for 5 min.

### Non-specificity of PP9-competitor interaction

This experiment was carried out to test whether the competitor-induced enhancement of TOL expression was limited to a few species or whether it was a general response to a variety of potential competitors. We carried out additional experiments using a Gram positive bacillus (*Lactobacillus* sp.) and a Gram negative coccus (*Deinococcus radiodurans*). About 10^6^ cells each of PP9 and *Lactobacillus* sp. PP9 and *Deinococcus radiodurans* were inoculated in Tris+BA medium and the BA degradation profile was monitored as described earlier.

## Supporting Information

Figure S1 HPLC Chromatogram overlap of three injections, 5 mM Benzyl alcohol in Tris Medium.
(0.10 MB TIF)
