# Supplementary figures and images for: Competition Triggers Plasmid-Mediated Enhancement of Substrate Utilisation in Pseudomonas putida
Source: PLoS One. 2009 Jun 26;4(6):e6065. doi: 10.1371/journal.pone.0006065 (PMC2698150; doi:10.1371/journal.pone.0006065)

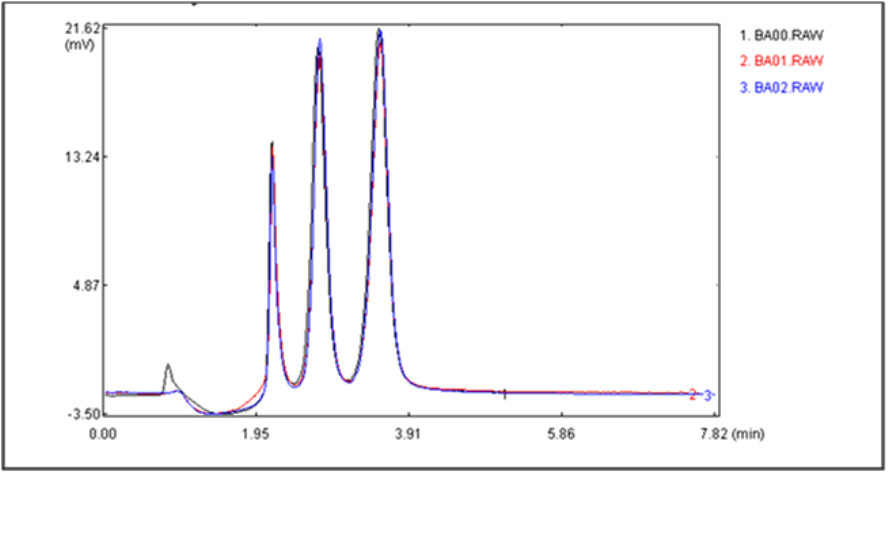

Supplement: Figure S1 — HPLC Chromatogram overlap of three injections, 5 mM Benzyl alcohol in Tris Medium. (0.10 MB TIF) [file pone.0006065.s001.tif]
